# Genomic Profile in a Non-Seminoma Testicular Germ-Cell Tumor Cohort Reveals a Potential Biomarker of Sensitivity to Platinum-Based Therapy

**DOI:** 10.3390/cancers14092065

**Published:** 2022-04-20

**Authors:** Rodrigo González-Barrios, Nicolás Alcaraz, Michel Montalvo-Casimiro, Alejandra Cervera, Cristian Arriaga-Canon, Paulina Munguia-Garza, Diego Hinojosa-Ugarte, Nora Sobrevilla-Moreno, Karla Torres-Arciga, Julia Mendoza-Perez, José Diaz-Chavez, Carlo Cesar Cortes-González, Clementina Castro-Hernández, Jorge Martínez-Cedillo, Ana Scavuzzo, Delia Pérez-Montiel, Miguel A. Jiménez-Ríos, Luis A. Herrera

**Affiliations:** 1 Unidad de Investigación Biomédica en Cáncer, Instituto Nacional de Cancerología-Instituto de Investigaciones Biomédicas, UNAM, Mexico City 14080, Mexico; rodrigop@ciencias.unam.mx (R.G.-B.); michelmontalvo@ciencias.unam.mx (M.M.-C.); carriagac@incan.edu.mx (C.A.-C.); paulina.munguia@anahuac.mx (P.M.-G.); karlamar@ciencias.unam.mx (K.T.-A.); jdiazchavez03@comunidad.unam.mx (J.D.-C.); ccortes@iibiomedicas.unam.mx (C.C.C.-G.); ccastroh6@unam.mx (C.C.-H.); 2 Novo Nordisk Foundation Center for Protein Research, Faculty of Health and Medical Sciences, University of Copenhagen, DK-2200 Copenhagen, Denmark; nicolas.alcaraz@cpr.ku.dk; 3 Instituto Nacional de Medicina Genómica, Mexico City 14610, Mexico; acerverat@inmegen.gob.mx; 4 Departamento de Cirugía, Hospital Regional de Alta Especialidad del Bajío, Leon 37660, Mexico; diego.hinojosa.ugarte@gmail.com; 5 Departamento de Oncología Médica, Clínica de Tumores Genitourinarios, Instituto Nacional de Cancerología, Mexico City 14080, Mexico; nsobrevillam@incan.edu.mx (N.S.-M.); jorge_martinezcedillo@yahoo.com.mx (J.M.-C.); 6 Department of Translational Molecular Pathology, The University of Texas MD Anderson Cancer Center, Houston, TX 77030, USA; JRMendoza@mdanderson.org; 7 Departamento de Urología, Instituto Nacional de Cancerología, Mexico City 14080, Mexico; ascavuzzo@incan.edu.mx (A.S.); mjimenezr@incan.edu.mx (M.A.J.-R.); 8 Departamento de Patología, Instituto Nacional de Cancerología, Mexico City 14080, Mexico; mperezm@incan.onmicrosoft.com

**Keywords:** TGCT, platinum-resistance, WES, CGH, SNVs, CNVs and DNA breakpoints

## Abstract

**Simple Summary:**

Despite having a favorable response to platinum-based chemotherapies, ~15% of Testicular Germ-Cell Tumor (TGCT) patients are platinum-resistant. Incidence and mortality of this disease has remained unchanged in Latin populations unlike the rest of the world. To date, the search for genetic variants in our population remains unexplored. The aim of this study is to identify predictive biomarkers of resistance to platinum-based therapy, whether general or specific to the Latin population. We observed that sensitivity to chemotherapy does not seem to be explained by any of the mutations detected. However, we identified amplifications on segment 2q11.1 as a novel variant with chemosensitivity biomarker potential. Our data shed light into understanding platinum resistance in a Latin-origin population.

**Abstract:**

Despite having a favorable response to platinum-based chemotherapies, ~15% of Testicular Germ-Cell Tumor (TGCT) patients are platinum-resistant. Mortality rates among Latin American countries have remained constant over time, which makes the study of this population of particular interest. To gain insight into this phenomenon, we conducted whole-exome sequencing, microarray-based comparative genomic hybridization, and copy number analysis of 32 tumors from a Mexican cohort, of which 18 were platinum-sensitive and 14 were platinum-resistant. We incorporated analyses of mutational burden, driver mutations, and SNV and CNV signatures. DNA breakpoints in genes were also investigated and might represent an interesting research opportunity. We observed that sensitivity to chemotherapy does not seem to be explained by any of the mutations detected. Instead, we uncovered CNVs, particularly amplifications on segment 2q11.1 as a novel variant with chemosensitivity biomarker potential. Our data shed light into understanding platinum resistance in a Latin-origin population.

## 1. Introduction

Testicular cancer accounts for approximately 1% of cancers in men; however, it is the most common tumor in males aged 15 to 44 [1]. Testicular germ-cell tumors (TGCT) comprise 98% of all malignant neoplasms that arise in the testicle. Based on histological analysis, TCGT can be classified as seminomas (SE-TGCT) and non-seminomas (NS-TGCT), both of which arise from germ-cell neoplasia in situ [2]. Seminomas encompass 50–60% of the tumors, with a peak incidence at age 35. Non-seminomas comprise about 40–50% and are usually diagnosed around 25 years of age, the latter are noted for their diverse histological subtypes and varying degrees of differentiation [3].

Since the introduction of platinum-based therapies, TGCTs have been widely recognized for their outstanding five-year survival rates. These values are close to 95% regardless of the histologic subtype and close to 80% for metastatic cancer [4]. Nevertheless, platinum refractory disease—which is defined as persistent or rising tumor markers during or within four weeks of completion of a four-cycle platinum-based chemotherapy regime—is observed in approximately 10–20% of patients. Further therapeutic options are limited for these patients, and long-term survival rates remain poor. Understanding this phenomenon has been of broad interest to clinicians; however, to date, the mechanism leading to platinum resistance is poorly understood. In addition, upfront identification of platinum-resistant or platinum-sensitive patients using biomarkers is not feasible in the clinical setting, and the development of targeted therapies seems to be far off [5].

Broadly, TGCTs are characterized by frequent chromosomal anomalies, with polyploidization and gain of chromosome arm 12p as an isochromosome being the most regularly observed. Gains of chromosome X, 7, 21, and 22 are also frequent in these tumors [6]. The mutation burden of this neoplasm has been described as low compared with that of other cancers; mutations in the *KIT* and *KRAS* genes have been highlighted as the most commonly reported driver genes for SE-TGCT [7].

To date, no mutations or genetic alterations have been described that could help identify resistance to platinum-based treatment. Several mechanisms, such as defects in homologous recombination, *TP53* and *PTEN* mutations, higher mutational burdens, and cell cycle regulation alterations, have been proposed as a possibility; however, none of them seem to adequately explain this phenomenon [8]. 

Recently, efforts have been made to elucidate the genomic characteristics that underlie the TGCT subtypes. Whole Exome Sequencing (WES) has been one of the most widely used approaches. Through this method, some differences were noted between platinum-resistant and platinum-sensitive tumors. For instance, the mutational burden of resistant tumors seems to be higher than that of sensitive tumors. New putative driver genes and pathways like WNT/CTNNB1 have been associated with resistance and *KIT* and *TP53* mutations were also significantly different from one another [9].

Incidence and especially mortality varies throughout different ethnicities, with non-Hispanic whites being the most commonly affected population in the US. Nevertheless, the Hispanic population is the second most affected group [10]. Recently, chemoresistance has been broadly studied in American non-Hispanic whites and European ancestry populations since they tend to have the highest incidence [9]. However, chemoresistance in Hispanics has not been well studied. Although disease incidence seems to have stabilized, mortality has behaved differently around the world [11]. Mortality in the US, Canada, and Europe have been decreasing, whereas in Africa, Asia, Latin America, and the Caribbean, mortality rates have remained stable or increased [11,12]. One recent study revealed that mortality in American populations of Hispanic origin showed a tendency to increase, independently of the stage at diagnosis and socioeconomic level [13], indicating differences in genetic susceptibility to TGCT at a population level. Although there is not enough evidence to suggest that the underlying chemoresistant mechanisms may be different between ethnicities, the divergence in mortality rates warrants studying the higher mortality populations separately. Taken together, this background indicates that there is an urgent need to explore these populations in order to improve early diagnosis and therapeutic approaches.

In this study, we carried out a genomic approach to broaden our understanding of TGCT platinum resistance in Hispanic populations by studying a Mexican cohort from the largest cancer center in Mexico using whole exome sequencing (WES) and microarray-based comparative genomic hybridization (aCGH) We observed a greater mutational load compared to that reported in other populations. We found mutated genes that are unusual and could characterize our NS-TGCT patients. Also, we examined changes in copy number variants (CNVs) in greater detail and identified amplification of a new region that could serve as a biomarker of sensitivity for platinum-based therapy. DNA breakpoints in genes were also explored, and interesting novel findings were revealed. 

## 2. Materials and Methods

### 2.1. TGCT Patient Cohort

For this study, 32 patients with clinicopathological diagnosis stage type II or III of NS-TGCT were selected; the patients were treated between 2008 and 2018 at the National Cancer Institute of Mexico (INCan). 

Patients underwent radical orchiectomy and were histopathologically classified according to the 2016 WHO Classification of Tumors of the Urinary System and Male Genital Organs. Patients were staged according to the American Joint Committee on Cancer (AJCC), 8th edition, and classified according to the prognostic groups of the International Collaborative Group of Germ-Cell Tumors (IGCCCG). All patients received treatment with bleomycin, etoposide, and cisplatin (BEP) as established by the NCCN Clinical Practice Guidelines in Oncology. After treatment, objective response rates were evaluated with CT scans within 6 weeks of conclusion of BEP chemotherapy based on RECIST criteria version 1.1. Platinum resistance was defined as a disease that continually progressed under platinum-based chemotherapy, progressive disease (relapse or incomplete response) after one or more complete platinum-based regimes within the first two years of follow-up, viable (non-teratomatous) disease in a post-chemotherapy surgical sample, or persistent or rising tumor markers four weeks after a complete four-cycle chemotherapy regime [9]. Follow-up and re-classification were last updated in January 2021. 

A summary of the study cohort is detailed in Table 1. The ethics committee and the research committee of INCan approved the study protocol (012/031/ICI) and (CEI/783/12). The research followed the tenets of the Helsinki Declaration based on approval by the hospital’s institutional review board and was conducted after obtaining the subject’s informed consent in accordance with institutional guidelines. 

### 2.2. Sample Collection and DNA Extraction for WES

In order to detect somatic variants and distinguish them from germline, we use peripheral blood samples to match each solid tumor in order to filter only those changes that are specific to the tumor and not germline variants. Tumor and peripheral blood samples were obtained before chemotherapy, at the time of radical orchiectomy of NS-TGCT patients. After the pathology department confirmed the histopathological diagnosis by evaluating the tissues, samples were frozen at −80 °C 30 min after surgery (cold ischemia time) in RNA stabilization solution tubes. The remnant tumoral tissues were routinely fixed in formalin and paraffin-embedded for subsequent histological examination. DNA was extracted from chemo-naïve frozen tumors and peripheral blood for sequencing using the DNeasy Blood & Tissue kit (Qiagen, Valencia, CA, USA) following the manufacturer’s specifications. The quality and integrity of the DNA was analyzed employing the TapeStation 2200 (Agilent Technologies, Santa Clara, CA, USA) according to the specifications established by the manufacturer.

### 2.3. Statistical Analyses

The clinical variables of the patients included in this study were analyzed using descriptive statistics. Inferential comparisons were performed by calculating the relative risk with their 95% confidence intervals. For the differences between groups, Student’s *t*-test or Mann–Whitney u-test was used depending on the nature of the data. Chi-Square and Fisher tests were used to compare the categorical variables. The alpha value was defined as *p* < 0.05. All statistical tests were two-tailed. 

### 2.4. Whole Exome Sequencing (WES) 

The complete DNA exomes of 32 paired tumor tissue (to see somatic changes) and peripheral blood samples (used as control in each case for germline variants) were sequenced. From which, 18 were platinum-sensitive and 14 platinum-resistant patients. Sequencing was performed using the HiSeq2500 Illumina platform, following the Illumina Nimblegen V3 protocol established at the Cancer Genomics Laboratory of the University of Texas MD Anderson Cancer Center (MDACC). The samples were sequenced at an average coverage of 100× for the tumor samples and 60× for the control samples.

### 2.5. Sequence Data Resources (WES)

#### 2.5.1. Preprocessing

Exome preprocessing was performed following GATK (version 3.7) best-practice guidelines [14]. For quality control analysis of reads, we used FastQC version 0.11.4. Reads were mapped to the human reference genome hg38 using BWA-MEM (version 0.7.12-r1039). Afterwards, duplicates were removed using Picard (version 2.18.13). Base scores were recalibrated using the BaseRecalibrator and ApplyBQSR tools within the GenomeAnalysis toolkit package to obtain a final set of analysis-ready bam files. Then, a panel of normals was assembled using the CreateSomaticPanelOfNormals (gatk-4.0.8.1) tool with all normal samples, which was subsequently used as input for somatic variant calling. 

#### 2.5.2. Somatic Variants (SNVs and Indels) Analysis

Somatic variants were called with Mutect2 (gatk-4.0.8.1) using tumor and control samples for each patient, the panel of normals, and the parameters’ minimum mapping quality of 50 and minimum base quality score of 26. Variants were filtered for contamination using FilterMutectCalls (gatk-4.0.8.1). Filtered variants were annotated and classified with the Funcotator (within gatk-4.0.8.1) tool. Analyses and plots of annotated filtered variants were produced using the maftools R package. Relevant variants/indels were visually inspected using the IGV browser.

#### 2.5.3. Somatic Copy Number Variations (SCNVs) Analysis

For the detection, classification, and segmentation of SCNVs, the toolkit CNVkit (v.0.9.8) was used. Previously, the R package PureCN was employed to infer ploidy by purity of tumors, using this information to adjust the calls in CNVkit [15]. To find significant recurrent CNVs in sensitive and resistant groups, GISTIC (version 2.0.22) [16] and maftools R packages were used to visualize data. 

### 2.6. Microarray-Based Comparative Genomic Hybridization (aCGH)

Array-CGH analysis was performed on 8 paired samples (4 platinum-resistant and 4 platinum-sensitive) with peripheral blood DNA as control of germline from our TGCT cohort considering the recommendations of orthogonal validation. We took a representative sample of the cohort (25% of the patients) using Agilent SurePrint G3 Human Genome CGH Microarray kit 4 × 180 K (Agilent Technologies, Santa Clara, CA, USA) targeting structural variations. DNA was extracted from chemo-naïve frozen tumor tissue (see above) from TGCT patients whose platinum response was evaluated previously. For DNA extraction, we used the DNeasy Blood & Tissue kit (Qiagen, Valencia, CA, USA). 

The quality and integrity of the DNA were analyzed by TapeStation 2200 (Agilent technologies, Santa Clara, CA, USA) according to the specifications established by the manufacturer. Results were analyzed using Agilent Genomic Workbench (version 6.5) and Agilent CytoGenomics (version 5.1.2.1) software with the following settings: ADM-2 as aberration algorithm, threshold of 6, and moving average of 2 Mb. The results are annotated according to Human Genome build 19 and include imbalances with at least three consecutive probes with abnormal log2 ratios. 

## 3. Results 

### 3.1. TCGT Mutation Burden Is Higher in Mexican Patients, but Does Not Define Platinum Resistance

We recruited a series of 32 non-seminoma TGCT cases comprising 14 platinum-resistant and 18 platinum-sensitive patients (Table 1). The majority had more than one metastatic site, with non-pulmonary visceral metastases being quite common. All pathology reports were reviewed based on the most recent 2016 WHO Classification of Tumors of the Urinary System and Male Genital Organs. As expected, the majority of our patients had a mixed component histology, and pure tumors were rare among our cohort. The subtypes and distribution of mixed tumors are shown in Table 1. We also included a pair of malignant transformations since they are quite unusual, and their particular resistance to chemotherapy has not been explored. All of our patients were classified according to their IGCCCG group, with a considerable proportion of them having poor prognosis from the beginning of their treatment. Partial response was the most common outcome in our cohort, which was expected since intermediate and poor prognostic patients were common and the presence of teratoma in the primary tumor was frequent. At the time of this analysis, 28.1% of our patients were dead and 15.6% were lost to follow-up.

**Figure 1:**
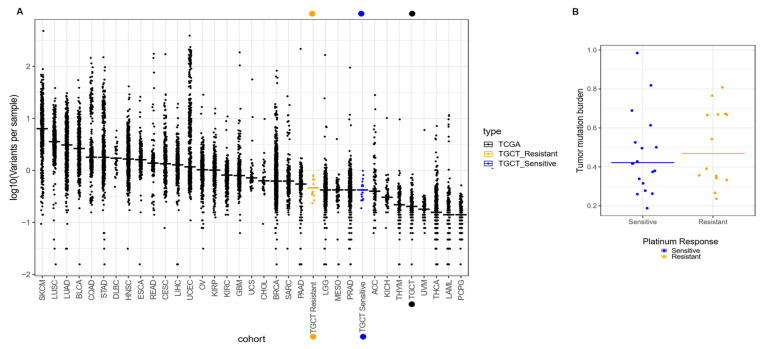
Comparison of tumor mutation burden in INCan-TCGT cohort vs. TCGA. (**A**) Median frequencies of somatic variants reported in exome sequencing (horizontal lines) across multiple tumor types reported in TCGA. Left to right, highest, and lower frequencies, as measured in mutations per megabase (Mb). Broadly, the INCan-TCGT cohort (orange and blue dots) has shown (both platinum sensitive and resistant samples) that the mutation rate is higher than reported in TCGA for TGCT tumors. (**B**) Discrimination of mutation burden by response. Platinum-resistant samples (orange dots) have shown higher (yet not significant) frequency in variants per sample in comparison with platinum-sensitive TGCT samples.

We extracted DNA from each patient from both frozen tumor tissue and peripheral blood as germline variant control, and performed WES assays, with a mean coverage of 100× across targeted bases, and did not compare our data with other populations. From the 32 cases, a total of 1100 variants were called (Figure S1), in which missense mutations were the most frequent (800 variants). Single nucleotide variants (SNVs) were the most commonly found. We identified around 900 different SNPs present throughout all patients. These were mostly composed of C > A and C > T transversions (1105 and 1890 SNV classes, respectively). As expected from previous reports, we observed a mean rate of 0.49 somatic mutations per Mb (Table 2). This represents a low mutation burden, when compared to other solid tumors like melanoma and lung cancer, which present rates of 11.0 and 8.0 mutations per Mb, respectively. In this regard, we wanted to determine if the mutation burden of our patients was similar to that reported in the TCGA database (0.31 mutations/Mb). In general, we observed that our patients had a mutation burden rate greater than the average TGCT previously reported, and such was the case even when we divided the patients into our therapy response groups (Figure 1A). TCGT-resistant and -sensitive patients had mutation burdens around 0.53 and 0.46 mutations/Mb, respectively. Although we observed a trend for a greater mutation burden in therapy-resistant than in -sensitive patients, there was not a significant difference (Figure 1B). Therefore, our data suggested that there was a higher mutation rate in our population when compared to the one reported in TCGA (mainly European ancestry population), but close to mutational rates reported more recently with sequencing coverages similar to 100×.

### 3.2. Gene Variants Are Not Sufficient to Characterize Response to Therapy

**Figure 2:**
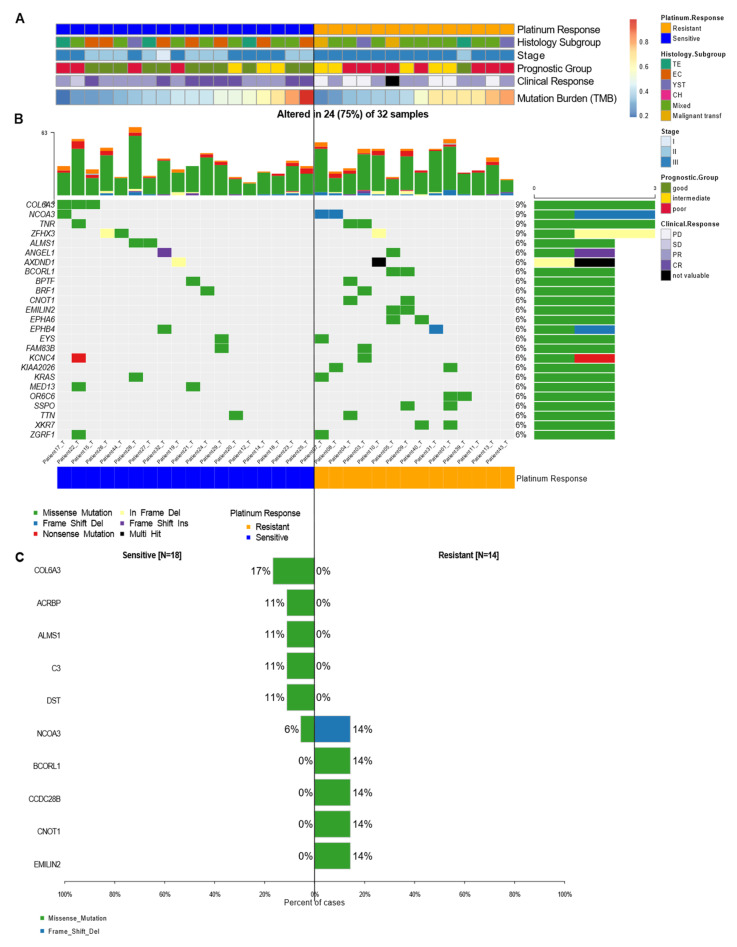
Mutational landscape of cancer driver genes in TGCT. (**A**) Heatmap with clinical features of patients, each row represents a sample divided by response group and another clinical characteristic. (**B**) Oncoplot showing the most frequently mutated coding genes across 32 NS-TGCTs by response groups. Each column represents a sample and each row a different gene with associated color for each mutation type. The top bar plot has the frequency and type of mutations for each patient, while the right barplot has the frequency of mutations in each gene. Samples are ordered by the platinum response group, resistant and sensitive (orange and blue, respectively). The highest frequency of mutations in each gene is 9% and represents all random mutations observed in at least two samples. (**C**) Most frequently mutated genes and percentage of cases where these mutations were found separated among groups, sensitive (left) and resistant (right). Neither gene significantly differentiates the groups.

To understand possible driver mutations, or those that could serve to identify therapeutic responses, we first explored the general features of our patients and their association with the clinical outcome, shown as clinical response and prognostic groups. As described in Table 1, we observed that our sensitive and resistant patients presented a heterogeneous histological subgroup, most of which was represented by the mixed type according to WHO guidelines for urological tumors, where EC and YST were the most common predominant components observed in mixed tumors. Moreover, most of the resistant patients were stage III and classified into the poor prognostic group. When analyzing their clinical response, we found either progressive or stable disease. On the other hand, 44% of sensitive patients who had predominantly good prognosis reached a complete response to therapy. Interestingly, the mutation burden was not directly associated with the prognostic group or TMB (Figure 2A). Regardless of the mutational burden, patients were still classified into the poor prognostic group, suggesting that mutation accumulation does not drive disease outcomes or the response to platinum-based therapy (Figure 2A). Next, we performed an analysis of the genes most significantly altered in all the patient samples based on their responses. Relevant variants/indels were also visually inspected using the IGV browser. 

Although we found *KRAS* mutations in 6% of the patients, this was not the most frequent mutation. In our samples, mutations affecting *COL6A3*, *NCOA3*, *TNR*, and *ZFHX3* were the most abundant (with 9% frequency each). In most of the genes, missense mutations were the most frequent type of mutation; however, some mutated genes such as *NCOA3*, *ZFHX3*, and *ANGEl1*, presented other varieties of mutations (Figure 2B). Taken together, these findings revealed that, except for the variants mentioned, most of the mutations seemed to be random and infrequent in the different response groups. This suggested that the mutations were not the direct cause of resistance to therapy, but they could lead to the risk of developing these types of tumors. 

Furthermore, statistical analysis using forest plots indicated that there were no significant differences between our groups (Figure 2C). Nonetheless, we managed to detect mutations in genes such as *COL6A3*, *ACBRBP*, *ALMS1*, *C3*, and *DST* (with a frequency between 17 and 11%) that were exclusively seen in sensitive patients. We also observed variants unique to platinum-resistant patients, such as *BCORL1*, *CCDC28B*, *CNOT1*, and *EMILIN2* (observed with a frequency of 14% each) (Figure 2C). These findings do not detract from the fact that these variants might possess biological significance. Of all the most frequent mutations, the *COL6A3* gene (9% frequency in NS-TGCT tumors) was the most representative one. Although this mutation was present only in sensitive patients, it was not suggested to be a significant sensitivity biomarker, but rather to be relevant as a driver gene for testicular cancer. Nonetheless, a larger cohort is required to validate this result. 

Taken together, our results showed, as expected, that platinum-resistant patients had a worse prognosis and clinical outcome. Our data suggested that the observed mutational variants are not capable of defining a response to platinum-based therapy. However, we identified different genes that could behave as potential drivers for NS-TGCT disease.

**Figure 3:**
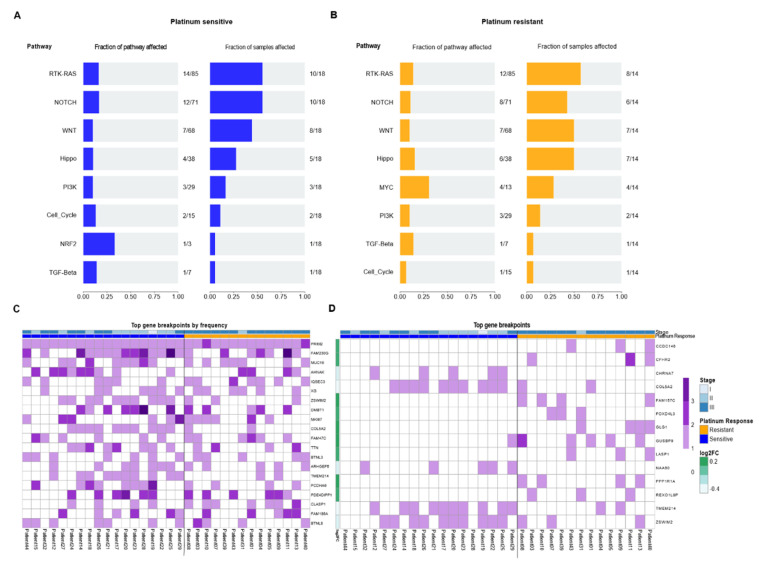
Oncopathways and genomic breakpoints analysis. (**A**,**B**) Frequency plot of oncopathways affected in platinum-sensitive and platinum-resistant samples, shown left to right, fraction of the pathway affected and the fraction of samples that present an event located in these pathways. There are not the same oncopathways affected between the two response groups. (**C**) Gene Breakpoint analysis, derived from CNVKIT: Top 20 most frequent genes of all the genes that had breakpoints in the whole cohort. (**D**) Oncoplot with most frequent breakpoints in genes that greatly separated the two conditions. The fold change is the log-ratio of the fraction of patients between resistant and sensitive that have those breakpoints. Log2 (fraction of breakpoints in resistant)/(fraction of breakpoints in sensitive).

Since individual genetic variants did not define response to therapy, we wanted to assess if the phenomenon was linked to specific cell pathways that could jointly distinguish sensitivity or resistance to treatment. We observed that there were pathways that were affected in more than 50% of patients, among which the most-affected were RTK-RAS, NOTCH, WNT, HIPPO, and PI3K. Specifically, the MYC pathway was more affected in resistant patients, while the NRF2 pathway was only identified in sensitive patients. However, as a whole, the affected pathways were independent of the response to platinum-based treatment. Thus, our results suggested that these pathways were tumor-dependent or specifically more affected in NS-TGCT patients, which could therefore suggest an indicator of risk for developing the illness (Figure 3A,B).

### 3.3. DNA Breakdown Sites Are Frequent and Differ in Groups of Patients with Different Responses to Platinum-Based Therapy 

It is known that chromosomal instability, especially DNA breaks, are of special relevance in cancer. We evaluated the frequency of genetic breakpoints that occurred in our patients. We observed that in most of our patients, many of these were concentrated in specific genes. This is the case for those present in *PRIM2*, *FAM230G*, and *MUC16* genes. This result seemed relevant to us since the mutations that we observed previously were random in nature, but the breakpoints were not. In fact, these breakpoints were demonstrated to be consistent hot spots in our patients. Some of these breakpoints had more than one cleavage site, such as those in the *FAM230G* and *AHNAK* genes (Figure 3C). Interestingly, all the patients presented breakpoints in the *PRIM2* gene. *PRIM2* is a regulatory subunit of the DNA primase complex and component of the DNA polymerase alpha complex, which is highly relevant for the initiation of DNA synthesis. Likewise, breaks in the *MUC16* gene were present in 50% of patients. *MUC16* is overexpressed in multiple cancers and plays an important role in tumorigenicity and acquired resistance to therapy. *MUC16* (usually referred to as *CA125*) is widely known and has been extensively used as a biomarker for ovarian cancer, and its expression has been associated with disease progression. 

Next, we wanted to determine if there were specific breaks among the clinical groups and their response to therapy. Therefore, we filtered them based on the log2 ratio between the frequency of breakpoints in the resistant cohort versus the sensitive one. We observed that breakpoints were more frequent in resistant patients, where the most constant breakpoints were those that occurred in *CCDC146*, *CFHR2FAM157C*, *GUSBP9*, and *PPP1R1A* genes. In therapy-sensitive patients, we found less frequent rupture sites (six genes) present in the *CHRNA7*, *COL5A2*, *NAA60*, *TMEM214*, and *ZSWIM2* genes (Figure 3D). These results suggested that DNA breaks occur at common sites in patients and that these breakpoints could be related to the phenomenon of chemoresistance. Therefore, this result suggested a panel of ruptures that are able to cluster and distinguish the response to platinum-based therapy in NS-TGCT patients, especially in Mexican populations. 

### 3.4. Copy Number Variation Analysis Could Define Differential Molecular Signatures for Platinum Sensitivity 

**Figure 4:**
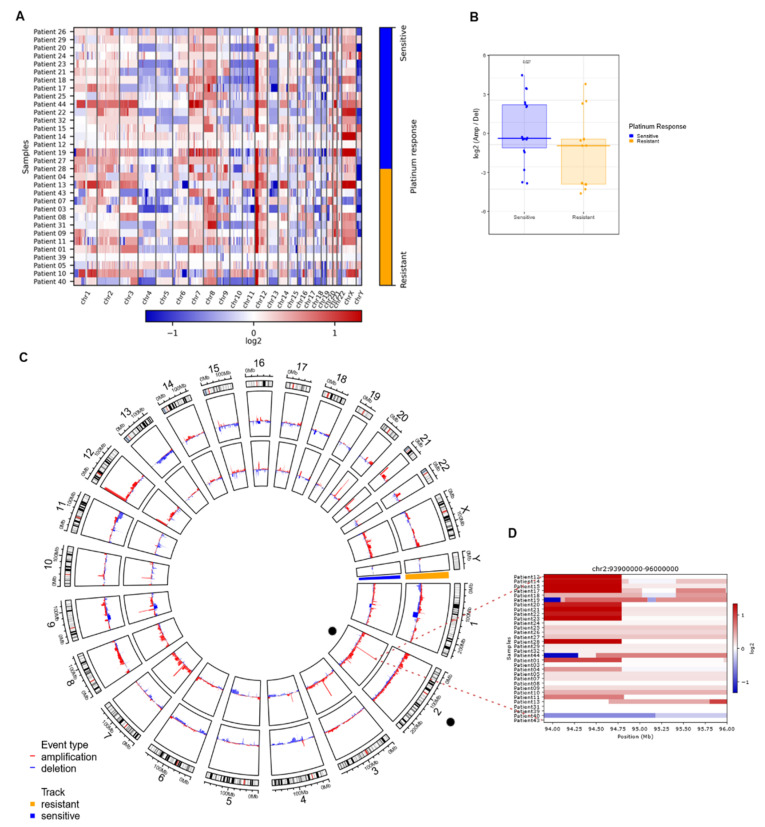
Significant CNVs segments that can distinguish between resistant and sensitive response to platinum. (**A**) Heatmap of calls in copy number variations (either bins or regions). It shows an overview of the larger-scale CNVs for all our samples (both platinum responses), bins of gain (red) and loss (blue) are in color range for all genome arrays showing low- and higher-amplitude segments. (**B**) Heatmap from CNVKIT focused on chr2, each row represents a patient sample, and each column represents an event (gain in red, loss in blue) that can occur in this position (Mb). Zoom shows a heatmap focused on events in the 2q11.1 region, gain events are mostly common in sensitive samples. (**C**) Circos plot of arm-level events obtained with GISTIC analysis, separating sensitive (inner circle, blue) from resistant (outer circle, orange). (**D**) Boxplot of log-ratio of amplifications over the losses, we see that the sensitive samples tend to have this ratio higher than the resistant ones (analysis exclude X, Y chromosomes, *p*-value from Wilcoxon test).

Copy number analysis was performed on the 32 primary TGCTs with complete clinical data using WES (Table 1). The absolute mean tumor purity was >40%. There was heterogeneity among the samples and a tendency for a higher purity on chemoresistant samples than chemosensitive samples (median = 0.54 and 0.44 respectively). Most tumors showed hyperploidy (72% had ploidy >2.5) (Table 2). With this data, the calls in the CNVkit tool for segmentation and detection of significant CNVs were adjusted with the ploidy information (Figure S2). Broadly, TGCTs exhibited an elevated rate of arm-level amplifications, the most frequently observed included gains of 12p (90%), 20q (62%), 2p (76%), 7p, and 8p (74%). The most frequent focal events were amplification of 12p13.31 (96%), 12p12.1 (93%), and 12p11.21 (92%) (Figure 4A), as well as deletion of 4q and 5q (67%), 10q (55%) and 11q (65%) 13p (40%), 18q (62%) and Y (90%). Additionally, we found around 29 amplification sites and 52 deletion sites (arm-level) distributed throughout the genome in all samples (Figure S3). 

To determine whether there were differences between the amplification/deletion events present in platinum-sensitive and platinum-resistant patients, we performed a Wilcoxon test on autosomal chromosomes of all samples, with the log-ratio of amplifications over losses. We observed that platinum-sensitive samples tended to have a significantly (*p* = 0.027) higher ratio than platinum-resistant samples. This suggested that platinum-sensitive samples have more amp/del events than resistant samples (Figure 4B).

Aiming to evaluate whether the differences between platinum-sensitive and platinum-resistant patients were focused on arm-level events, we made a multifactorial adjusted analysis on the frequencies of global, individual arm, or focal chromosomal aberrations (Figure 4C). We found important differences in focal CNV variation sites among both response groups, reported as false discovery rate corrected (G-Score value), estimated for genomic segments.

In order to assess if any of these alterations in CNVs were able to distinguish chemoresponse, we performed linear modeling with limma on the actual changes in copy number values as defined by GISTIC to identify significant gains or losses in arm bands between the resistant and sensitive groups. We found 2q11.1 to be the only significant aberration (adjusted *p* = 0.03) (Figure 4C). Figure 4D shows a heatmap of the chromosome 2 genomic region and the q.11.1 sub-arm region, which highlights the difference between the frequency of gains among sensitive and resistant samples. Our results suggested that a higher number of gain events in the region was strongly correlated with platinum sensitivity. Therefore, we suggest that focal gains in segment q11.1 of chr2 could be a biomarker of sensitivity to platinum-based therapy.

We found important differences in focal CNV variation sites among both response groups, reported as false discovery rate corrected (G-Score value), estimated for genomic segments. Broadly, G-Scores values by frequent segments indicated that resistant samples showed less variation in terms of broad events (Figure S3) than sensitive ones. Platinum-sensitive samples showed a greater number of events for both gains and losses, accentuating a higher density in gain events in regions where platinum-resistance remained unchanged.

Moreover, platinum-resistant tumors (Figure 4C) showed an increased frequency of gains in 12p13.31 compared to platinum-sensitive tumors. Also, our results showed certain regions with different gain or loss events between sensitive and resistant tumors, but for the most part, events in various segments were repeated between both response groups (Figure S3).

**Figure 5:**
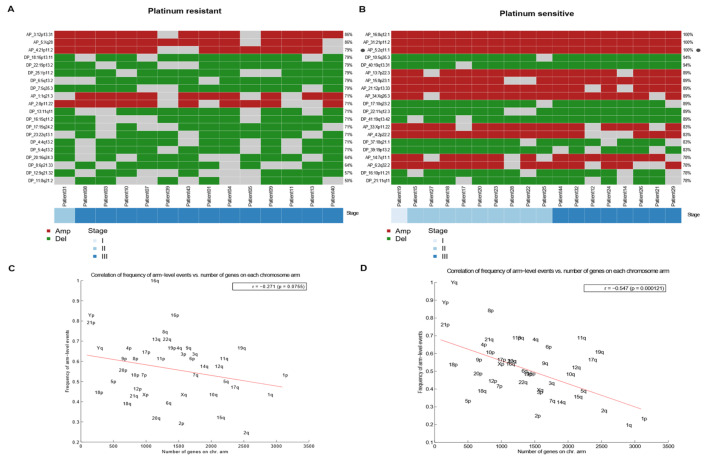
Most frequent arm-level amplifications/deletions events between sensitive and resistant TGCT. (**A**,**B**) Heatmap of arm-level regions top 20 in platinum resistant samples, each row represents a segment with its frequency, and each column a patient of the cohort that presents an event in these regions. Ordered by stage. Left to the right, resistant and sensitive. (**C**,**D**) Correlation (with significant *p* value) between frequency of arm level events (Y) and number of genes in chromosome arm (X) in platinum-resistant and platinum-sensitive, respectively. Anticorrelation (-r) is higher in sensitive samples, left to the right, resistant and sensitive.

To test this model, we evaluated possible differences between the response groups through the most aberrant copy number analysis. We determined the top 20 recurrent focal-level CNVs according to the GISTIC analysis for only resistant samples (Figure 5A) and for sensitive samples (Figure 5B). The results showed an important difference between the amplification and deletion events. Platinum-sensitive samples had a greater number of amplification events than platinum-resistant ones, which seem to have mostly deletion events. In resistant samples, the most frequent amplification segment was 12p13.31 (86%) (Figure 5A), whereas in sensitive samples, amplification of 8q12.1 was present in 100% of patients (Figure 5B). Furthermore, it is important to note that the difference between the amp/del events between both response groups did not seem to be conditioned to late stages of cancer, since in both early and advanced stages, the frequency of amp/del seems to stay the same. 

To evaluate whether the most frequent loss or gain events were limited to areas where there was a higher density of genes, we performed an anti-correlation analysis that showed that a higher density of genes implied fewer loss/gain events (Figure 5). Resistant samples tended to have higher losses/gains in gene-dense areas (Figure 5C) than platinum-sensitive samples. Conversely, the anti-correlation was more pronounced in platinum-sensitive samples (Figure 5D), which revealed that they tended to have a greater number of loss/gain events in areas of lower gene density than do those that were platinum-resistant. 

### 3.5. Array-CGH Validates Copy Number Variations on Genomic Regions Finding in WES

**Figure 6:**
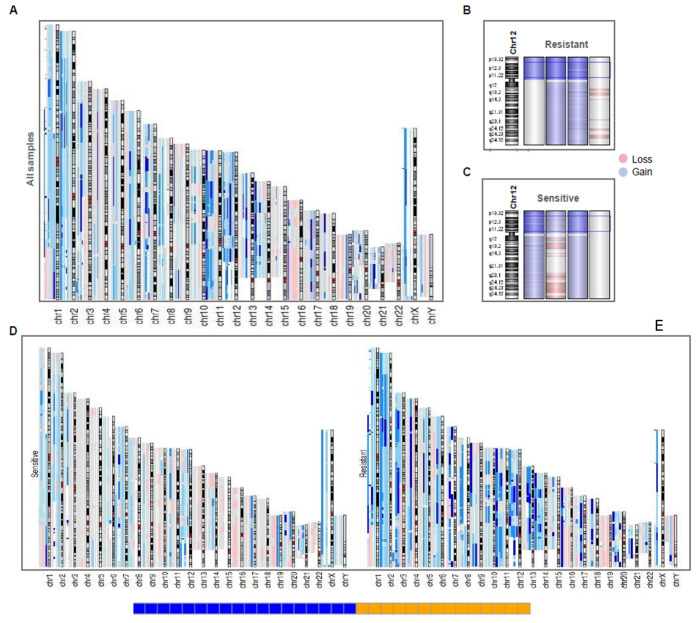
CGH array reveals a genomic landscape of instability in TGCT patients. (**A**) aCGH of all samples, each sample with large events in the chromosome array was aligned in the same position. Deep color in a region represents a higher frequency of incidence (two or more samples) of events in the region (gain in blue, loss in red). (**B**,**C**) Resistant (up) and Sensitive (down) samples in chromosome 12, showing that sensitive and resistant ones had 12p amplification, except in a case of each group. (**D**,**E**) Resistant and Sensitive ones aligned separately to view the differences on frequency of gain and loss events, showing that resistant ones present more events of gain than sensitive samples.

According to previous exome studies, the gold standard for the validation of copy number variation data in WES analysis is CGH array, following recommendations of orthogonal validation, on 25% of the patients (Figure 6). Therefore, to confirm our previous results, we performed a genomic hybridization microarray. Comparison of Agilent SurePrint G3 with 4 × 180 K hybridization probes focused on the search for CNVs, in four platinum-sensitive tumors and four platinum-resistant tumors paired with reference DNA from each individual belonging to the same cohort sequenced by WES. CNV call profiles were constructed individually for each sample, finding sites with more informative CNVs for each probe on all chromosomal arrays. CGH array also showed a variation in CNV-significant calls per sample in most resistant cases, which were reported as >50 (i.e., there were >50 bins or regions with CNVs events, either Amp, Del, Gain, or Loss). These results should be able to distinguish between the two platinum response groups and could define molecular signatures based on frequent CNVs. The concordance for CNVs between the exome analysis and the aCGH was >80% according to the average of amplifications and deletions, as was the concordance between the focal events compared with the segmentation of the GISTIC and CNVKIT tools used in the WES analysis.

As expected, the aCGH revealed a general landscape of genomic instability with more frequent events of gains rather than losses (Figure 6A). Broadly, all variation sites shown in WES (GISTIC analysis) were also found in aCGH due to genomic hybridization deep-recovery probes used (4 × 180 K). For instance, gains of chrX and losses of chrY were consistent in all samples (both previously reported in literature). This did not occur with isochromosome 12p amplification, which was not found in a sensitive sample. However, these results were consistent with the WES analysis, since both samples did not show the i12p amplification (Figure 6B,C).

Also, aCGH revealed a general landscape in which resistant samples presented more amplification events compared to sensitive samples (Figure 6D,E). This suggests that resistant patients had higher genomic instability than platinum-sensitive tumors. Finally, we showed chromosome 2 and the amplified segment 2q11.1 in a platinum-resistant sample (Figure S4A) and a platinum-sensitive (Figure S4B) sample to compare them with the relationship found previously, where the gain events in the region seemed to define the innate sensitivity of the patients. The occurrence of this event was shown by an opening in the region, where the genes that make up the region were laid out with their respective gain events.

## 4. Discussion

Testicular cancer is known to have a low incidence when compared to other neoplasms; however, its incidence is quite variable when comparing different regions. Incidence of germ-cell tumors in Latin America, as well as in Mexico, is not well established. Our hospital is a high-reference center for these tumors, accounting for up to 25% of urological cancers, just below prostate cancer [3,17]. Worldwide, mortality rates are low and in the last few years they have stabilized or decreased in several countries. This has been attributed to better screening techniques, prompt diagnosis, and the widely recognized sensitivity to platinum-based chemotherapy. However, for Mexican and Hispanic origin populations, mortality rates have increased [11]. Reasons for the increase in rates could be related to differences in genetic susceptibility to TGCT and chemoresponse mechanisms. However, to date, most of the genomic studies of TGCT have focused on characterizing the disease specifically by clinical risk factors, and have not focused on elucidating resistance to chemotherapy, which remains an unresolved clinical issue [18,19,20]. Previous genomic studies have aimed to unravel this phenomenon, but unfortunately, few non-European ancestry samples have been included in these studies. Hispanic populations have not been brought upon and remain to be broadly understudied. To our knowledge, we have assembled the largest NS-TCGT WES series from Hispanic-Mexican patients to date, also, we identified a series of novel mutated genes that have not been described and may be of relevance for Latin American populations [6,7,9].

In concordance with previous studies, we observed that NS-TGCT patients have a low mutation rate compared to other tumors [6,7]. The Shen et al. 2018 study (USA) contains the largest cohort to date reported in TCGA with a TMB calculated of 0.31 mutations/Mb (mean sequencing depth of ~20×); however other UK studies with higher depths (~72×) reported a higher TMB (0.51/Mb) [6,7]. On the other hand, a more recent study [9] included all available exomes from previous studies with heterogeneous depths (~20×–130×), obtaining a TMB of non-synonymous mutations of 0.35/Mb. In contrast, the mutation load that we report (0.49/Mb) is closer and slightly higher than the most recent UK reports [6,9]. The difference of our TMB between TCGA and INCan (Mexico) cohort, could well be due to the fact that we presented a greater depth, closer to UK studies (~97×) than the TCGA cohort, where the TMB could have been underestimated due to the low depth of sequencing [7]. The heterogeneity in standard methods of quantification and the lack of a robust and universal cutoff to identify biomarkers represents one of the main limitations for adopting TMB as a biomarker in clinical practice, which is why a need has been detected to standardize TMB reports in the cancer genomics community [21].

Taking these into account, we observed according to earlier reports that this rate was slightly higher in patients resistant to platinum-based therapy than those that are sensitive [9,22]. Although advanced stages are usually correlated with higher TMB in other cancers, previous reports of TMB in NS-TGCT had not considered the stage as a relevant variable for mutation burden. However, a recent study demonstrated that in fact, the TMB increases (not significantly) in metastatic (resistant all of them) vs. primary tumors, regardless of stage [9]. Just as in this study, the main difference was observed to occur between chemoresponse groups, where resistant patients have higher mutation rates than sensitive ones, regardless of being early or advanced stages.

Interestingly, although we detected mutations in *KRAS*, these were not the most abundant in our cohort. A frequency of 6% was observed when analyzing *KRAS*, differing from results in other populations where it has been as high as 12% [7,9,23]. When compared to other studies mainly focused on European ancestry populations, the most frequent set of mutations that we found, headed by the *COL6A3*, *NCOA3*, and *TNR* genes, are not usually reported in the top 20 variants. Additionally, we did not find mutations in the *KIT* or *TP53* genes, which have been described as frequent in TGCT. A recent study observed that although *KIT* has frequent mutations, they are mainly present in SE-TGCT tumors, which explains why it was absent, since our population was mainly NS-TGCT [6,23]. Interestingly, the most frequently mutated genes (Figure 2B) in our cohort are not reported yet in public databases as genes with the highest frequency. Even some of the genes that we expected to find with higher frequencies were not found higher than 6% (*KRAS*). So, although our main findings were not shared with public datasets (possibly due to population genomic differences), it is well known that for NS-TGCT, tumor-representative gene variants, such as *KIT* variants found only in seminomas, have not been found. This could be explained by the mutation frequencies of certain oncogenes not being as high in TGCT (up to 2–6%) [6,7,9]. Taken together, our data suggest that both the mutational burden and the mutational profile could be different in patients of Mexican origin.

Some studies refer to mutations in *TP53* as well as alterations in *MDM2* as characteristic of platinum-based resistance phenotype and an indicator of poor prognosis [23]. This was not the case in our study since the variants were not among our results. Although the variants were not significantly able to clearly define resistance or sensitivity to platinum, we detected those that showed a tendency to be specific for sensitivity (*COLA6*, *ACBRBP*, *ALMS1*, *C3*, and *DST*), with frequencies between 17 and 11% in these tumors, and those with a tendency toward platinum resistance (*BCORL1*, *CCDC28B*, *CNOT1*, and *EMILIN2*) that presented a frequency of 14%. We suggest that they may be considered potential biomarkers that require a larger study for their validation and future use in the clinical setting.

Interestingly, we revealed that individual genetic variants do not appear to define the response to platinum-based therapy in NS-TGCT patients. However, variants frequently occurred in eight main molecular pathways, headed by RTK-RAS, NOTCH, and WNT; these have at least one or two mutated genes present in the pathway. Although they do not define resistance, it seems that they were the main pathways involved in the development of NS-TGCT in our population. In contrast, studies focused mainly on European ancestry populations found RAS-RAF, PI3K/MTOR, and WNT/CTNNB1 as the mainly affected pathways which highlights the need to study Hispanic populations [7,9]. Nevertheless, more studies will be required to understand the importance of these variants as risk biomarkers for the disease. 

It is known that these tumors present clearer indicators of copy number and structural variations. We addressed the possibility that the presence of DNA breakpoints could be relevant in the etiology of the tumor and in the response to platinum-based therapy [24]. Remarkably, we observed the presence of DNA breakpoints that were characteristic of NS-TGCT independently of the response to therapy and those that could serve as a panel to distinguish between patients from the sensitive and resistant cohort.

There is currently only one report that studies breakpoints in TGCT [24]; however, the breakpoint gene set is not shared with the previous study. In *PRIM2* and *MUC16* genes with breakpoints in the major part of the cohort, we found only frequencies reported for SNVs in the TCGA cohort, where *MUC16* had 4.5% and *PRIM2* had 0.6%. To our knowledge, this was the first time that this has been reported and observed in the Hispanic population. The relevance of these breakpoints for their use in the clinical setting is a new field to explore in future research. 

Structural and CNV variations have recently gained importance in the setting of describing TGCTs. As previously described, we found that TGCTs are mostly characterized by structural aberrations since the development of the disease, probably due to early non-disjunction of Primordial Germ Cells that lead to amplifications and deletions of both focal and arm-level chromosomal regions like the 12p isochromosome [25]. There is no evidence of the second step of polyploidization, as in the i12 case. Nevertheless, microduplication events are frequent in TGCTs and germline cancers. 

Overall, we found that the high frequency of large-arm CNV events in NS-TGCTs does seem to be different between sensitive and resistant patients in accordance with previous studies [9]. However, CNVs analysis revealed that genomic instability in sensitive patients is slightly higher than in resistant patients, with a clear pattern in some focal amplifications with high copy number states as gains in 8q12.1 and 21p11.2. Particularly, gains of chromosome arm 2q11.1 are present in 100% of sensitive NS-TGCT, and it was significantly associated with chemosensitivity. Recently, the association between different loci and predispositions and TGCT heritability have become relevant. The most complete effort in this regard [26] included 22 loci with a high relationship in the susceptibility of TGCT. The 2q11.1 segment did not appear in the list of locus. At arm-level, recurrent amplifications of 2q11 have not been previously reported in TGCT [26], but it has been previously associated with neurological disorders [27,28]. Also, gains in 2q11.1–q11.2 were found with high frequency in Undifferentiated Pleomorphic Sarcomas, but its clinical relevance was not reported [29]. Specifically, in surrounding regions, gains on 2q11.2 have been found through CGH array in malignant peripheral nerve sheath tumors [28], losses in 2q11.2 also were found in follicular lymphoma [30], although their clinical associations were not reported. Currently, efforts are being made to integrate structural variations at a chromosomal level as a possible main driver in the development of TGCT [8,26]; however, there are no previous reports of focal aberrations as biomarkers of response to platinum-based therapy, where it is most likely a multifactorial phenomenon.

Our results are not enough to explain which mechanisms are leading to chemoresponse, but they coincide with recent efforts, where it was observed that mutational variants by themselves could not be the underlying cause of this phenomenon [7,8,9]. Taken together, the prevalence of increased mortality rates in Hispanic origin populations and the lack of chemo-response biomarkers, suggest that response to platinum-based chemotherapy is most likely of multifactorial origin (although a major, still unidentified driver alteration is possible) and further studies are required [8]. Although several efforts have been made, the role of focal or arm-level CNVs for the chemoresponse mechanisms is not yet clear. In our study, the high frequency of gain/losses in platinum-sensitive patients and the overall low TMB suggested that CN events could be more associated with the development of platinum-sensitivity. Some reports also suggested that rather than a mere passenger effect, the focal amplifications may reflect an active alternative mechanism by which tumor cell death is promoted and sensitizes tumor cells to therapy [31,32]. In this context, the focal amplification of 2q11.1 as a promising biomarker could open new paradigms to investigate CNVs as the primary cause of the development of chemoresponse mechanisms.

Finally, our findings are consistent with previous works focused on TGCT-CNV analysis. However, we found a higher frequency of gain or loss events in the sensitive patients, than the resistant ones, which is discordant to other populations studied [9]. Such discordances between sensitive and resistant patients could be due to variations in the ploidy and tumor purity of our cohort. Therefore, we made an exome validation through the CGH-array. Considering the guidelines of orthogonal validation, we took a representative sample of eight patients of the WES-cohort (25%). Taking into account the conditions that they presented in the results by WES, we chose the most interesting cases that we found by exome in order to validate our results regarding changes in CNVs and chromosomes, finding an overall correlation of 80% between WES and aCGH cases and up to 90% per case. Due to the high correlation observed per case (>90%), we can assume that our main findings are sufficiently valid. With the aCGH validation, even the focal events with the lowest frequency in CNVs barely detected by WES (which could not be informative by themselves), helped us to confirm that CNVs and structural variations are more common in sensitive patients. In addition, the aCGH replicated the finding of 2q11.1 amplification in all sensitive tumors from CGH array (Figure S4), confirming that their study could be replicable and potentially purposed for clinical use. Notwithstanding our principal limitation is the size of our cohort due to the low frequency of NS-TGCTs platinum-resistant patient availability, in this regard, another study with more samples, also suggested predominance of molecular signatures with inverse association with platinum resistance [9].

In our study, we demonstrate throughout our results how there are significant differences in the various parameters studied (CNVs and breakpoints). This shows that we cannot assume that the results of other populations such as European ancestry are the only relevant ones or that they are representative for all other populations. Taken together, our results show an advance in the characterization and genomic exploration of NS-TGCT in a population of Latin origin. Suggesting that even with similar methodological processes and applied treatments, there is a heterogeneity among the genome of different populations, which strengthens the need to explore larger Latin populations. 

## 5. Conclusions

Our results showed that the NS-TGCT cohort of Mexican patients analyzed by WES presented a higher mutational burden than what has been reported internationally in TCGA for testicular germ-cell tumors. The mutation burden in platinum-resistant patients shows a tendency to be higher than the one of platinum-sensitive patients. However, we did not find high-frequency mutated genes that are related to oncopathways or DNA-repair. Therefore, we suggest that in our cohort, the acquisition of resistance is not linked to an increase in mutations on oncodriver genes. In addition, we found a high frequency of breakpoints in relevant genes throughout the entire cohort, some of which could serve as a potential panel to differentiate between the two platinum response groups. They could also define the principle of genomic instability or the etiology in NS-TGCTs.

Our data revealed that our cohort presents genomic instability, characterized by a wide list of altered segments throughout the genome. Moreover, higher CNV events were found in platinum-sensitive patients than platinum-resistant ones. Finally, we found that gains in the 2q11.1 segment significantly distinguished platinum-sensitive from platinum-resistant patients; therefore, this could be considered as a potential biomarker of sensitivity to platinum-based chemotherapy in Latin-origin patients.

## Figures and Tables

**Table 1:** Clinical features of Testicular Germ-Cell Tumors.

**Response to CT, (*n*, %)**	**NS-TGCT Cohort (*n* = 32)**		
	**Resistant (*n* = 14)**	**Sensitive (*n* = 18)**	**All samples (*n* = 32)**
	14 (43.75)	18 (56.25)	
**Age at diagnosis, years: median, (IQR)**	25 (21–31)	22 (19–24)	24 (21–31)
	*p* = 0.13		
**Stage according to TNM from AJCC 8th Edition (*n*, %)**			
IS	0	1 (5.56)	1 (3.13)
II	1 (7.14)	9 (50)	10 (31.25)
III	13 (92.86)	8 (44.44)	21 (65.63)
	*p*″ = 0.008 *		
**Histologic Subgroup (*n*, %)**			
Embryonal carcinoma (EC)	10 (71.43)	14 (77.78)	24 (75)
Teratoma (TE)	9 (64.29)	13 (72.22)	22 (68.75)
Yolk Sac Tumors (YST)	10 (71.43)	13 (72.22)	23 (71.88)
Choriocarcinoma (CH)	2 (14.29)	2 (11.11)	4 (12.5)
Seminoma cells (SE)	6 (42.86)	2 (11.11)	8 (25)
Malignant transformation (MT)	2 (14.29)	0	2 (6.25)
Mixed histology **	13 (92.86)	17 (94.44)	30 (93.75)
Metastases (*n*, %) *			
Lung	11 (78.57)	6 (33.33)	15 (46.88)
**Lymph Node**			
Mediastinum (MLN)	4 (28.57)	2 (11.11)	6 (18.75)
Retroperitoneum (RPLN)	14 (100)	14 (77.78)	26 (87.5)
Inguinal (ILN)	2 (14.29)	3 (16.67)	5 (15.63)
**Non pulmonary visceral metastases**			
Liver	4 (28.57)	1 (5.56)	4 (12.5)
Bone	1 (7.14)	0	1 (3.13)
Brain (CNS)	2 (14.29)	0	2 (6.25)
No metastatic site	0	2 (14.29)	2 (6.25)
	*p* = <0.001 *		30/32 (93.75%)
**Prognostic group according to IGCCCG (*n*, %)**			
Good	1 (7.14)	11 (61.11)	12 (37.5)
Intermediate	5 (35.71)	3 (16.67)	8 (25)
Poor	8 (57.14)	4 (22.22)	12 (37.5)
	*p*′ = 0.01 *		
CT Regimen *** (*n*, %)			
BEP	1/14 (7.14)	18/18 (100)	19/32 (59.38)
BEP-RT	1/14 (7.14)	0/18	1/32 (3.13)
BEP-TIP	4/14 (28.57)	0/18	4/32 (12.5)
BEP-TIP + CISCA	5/14 (35.71)	0/18	5/32 (15.63)
BEP-CISCA	1/14 (7.14)	0/18	1/32 (3.13)
BEP-Doxorubicin	1/14 (7.14)	0/18	1/32 (3.13)
EP-VeIP-CISCA	1/14 (7.14)	0/18	1/32 (3.13)
**Response to platinum-based chemotherapy according to the RECIST version 1.1 (*n*, %)**			
Complete Response (CR)	0	8 (44.44)	8 (25)
Partial Response (PR)	7 (50)	9 (50)	16 (50)
**Overall Response Rate (ORR)**	7 (50)	18 (100)	25 (78.13)
Stable Disease (SD)	0	1 (5.56)	1 (3.13)
Progressive Disease (PD)	6 (42.86)	0	6 (18.75)
Not Evaluable (NE)	1 (7.14)	0	1 (3.13)
	*p*″ = 0.01 *		
**Outcome (*n*, %)**			
Alive	2 (14.29)	16 (88.89)	18 (56.25)
Death	8 (57.14)	1 (5.56)	9 (28.13)
Unknown	4 (28.57)	1 (5.56)	5 (15.63)
	*p*″ *=* 0.002 *		

Abbreviations: **CT**, chemotherapy; **BEP**, Bleomycin/Etoposide/Cisplatin; **IQR**, Interquartile Range; **TNM**, Tumor/Node/Metastasis; **MLN**, Mediastinum lymph Nodes; **RPLN**, Retroperitoneum Lymph Nodes; **ILN**, Lymph Nodes; **AJCC**, American Joint Committee on Cancer; **RT**, Radiotherapy; **TIP**, Paclitaxel/Ifosfamide/Cisplatin; **CISCA**, Cisplatin/Cyclophosphamide/Adriamycin; VeIP, Vinblastine/Ifosfamide/Cisplatin; **EP**, Etoposide/Cisplatin; IGCCCG, International Germ-Cell Cancer Collaborative Group; **RECIST**, Response Evaluation Criteria In Solid Tumors v1.1. *p*: Student’s *t*-test Sensitive versus Resistant. *p*′: Mann–Whitney´s U-test frequency of metastatic site per patient, Sensitive versus Resistant. *p*″: Fisher’s exact/Chi-squared test Sensitive versus Resistant. * Patients that presented metastases in that site at time of diagnosis. ** Mixed histology when 2 or more histological subtypes were identified according to the 2016 WHO Classification of Tumors of the Urinary System and Male Genital Organs. *** 2 to 4 platinum-based chemotherapy.

**Table 2:** Genomic alterations summary according to CT response of NS- TGCT cohort.

**Response to CT (*n*, %)**		**NS-TGCT Cohort (*n* = 32)**		
		**Resistant (*n* = 14)**	**Sensitive (*n* = 18)**	**All samples (*n* = 32)**
		14 (43.75)	18 (56.25)	24 (16–36)
Genomic alterations				
Somatic Variants (SNV)	**Mutation rate/Mutations per Mb, median, (IQR)**			
	Tumor Mutation Burden (TMB)	0.53 (0.4–0.7)	0.46 (0.3–0.6)	0.49 (0.3–0.7)
	Standard Deviation	0.19	0.21	0.20
		*p* = 0.36		
Copy Number variations (CNV)	**Tumor Ploidy and Purity, mean, (IQR)**			
	Ploidy (Absolute)	3.11 (2.4–4.0)	2.78 (2.2–3.0)	2.92 (2.4–3.5)
	Standard Deviation	0.98	0.57	0.77
		*p*′ = 0.17		
	Purity (Absolute)	0.54 (0.4–0.7)	0.44 (0.3–0.6)	0.48 (0.4–0.6)
	Standard Deviation	0.21	0.16	0.75
		*p* = 0.13		

*p*: Student’s *t*-test Sensitive versus Resistant. *p*′: Mann–Whitney’s U-test Sensitive versus Resistant.

## Data Availability

The exome sequencing and array-based data generated in this study have been submitted to the EGA database (https://ega-archive.org, accessed on 16 April 2022) under submission number EGAS00001006216.

## Supplementary Materials

The following supporting information can be downloaded at: https://www.mdpi.com/article/10.3390/cancers14092065/s1, Figure S1: Summary of coding somatic variants found on NS-TGCT cohort; Figure S2: Comparison of tumor ploidy and purity median from the sequenced samples obtained by PureCN analyses; Figure S3: Comparison of amp/del per segment over resistant (up) and sensitive (down) from GISTIC analysis; Figure S4: Visualization of aCGH in Chr2 region in two samples to compare focal-CNVs inl 2q11.1 segment.
