# Supplementary material for: Genomic Profile in a Non-Seminoma Testicular Germ-Cell Tumor Cohort Reveals a Potential Biomarker of Sensitivity to Platinum-Based Therapy
Source: Cancers (Basel). 2022 Apr 20;14(9):2065. doi: 10.3390/cancers14092065 (PMC9101377; doi:10.3390/cancers14092065)
Supplement: Supplementary file 1 [file cancers-14-02065-s001.zip › cancers-1652702-supplementary.pdf]

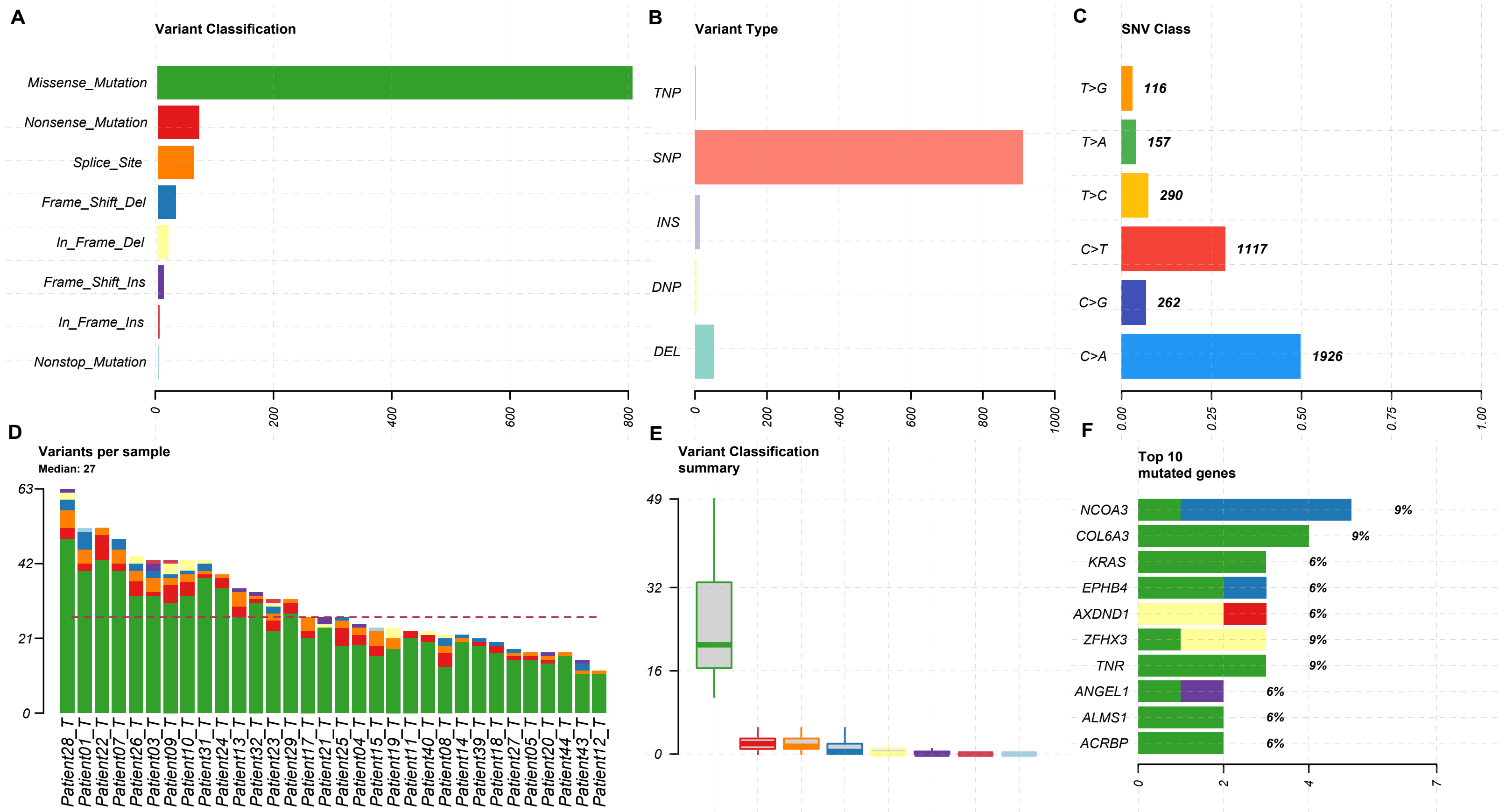

Figure S1. Summary of coding somatic variants found on NS-TGCT cohort. **(A)** Frequency and classification of variants found (missense). **(B)** Types of mutational variants most frequently found (SNP). **(C)** Transversions and transitions represented in the cohort **(D)** Variants found per sample (median 27 variants). **(E)** Distribution of variant types found per sample (color scheme is the same as a). **(F)** Top 10 genes mutated on the cohort and mutation types.

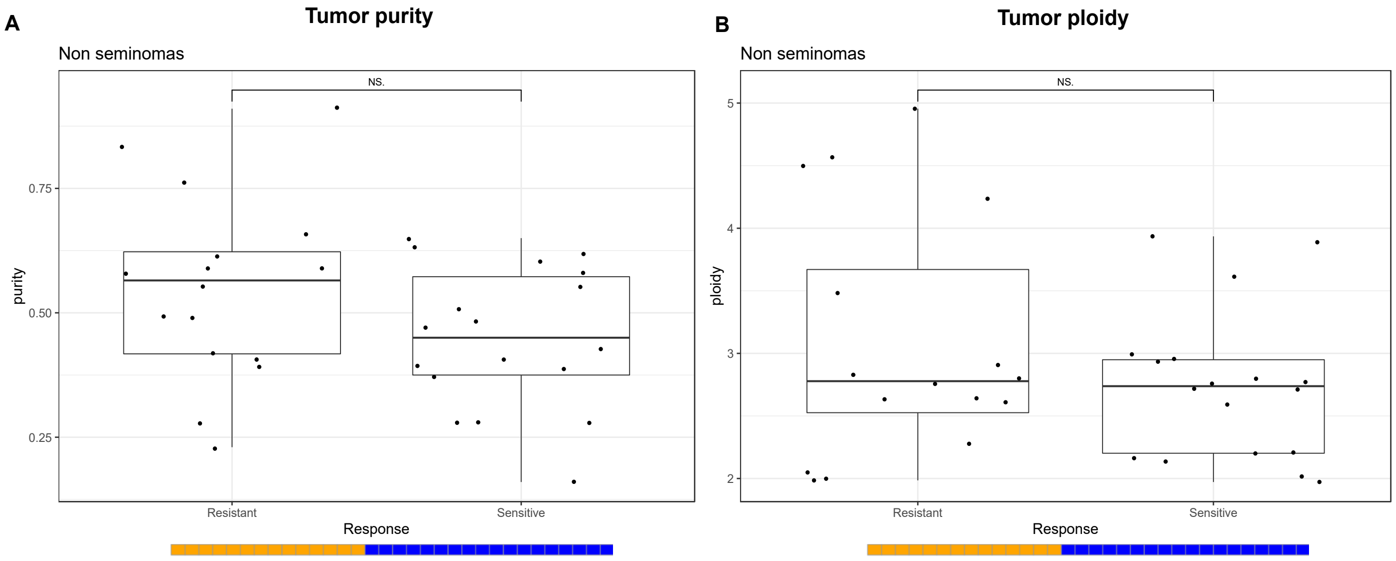

Figure S2. Comparison of tumor ploidy and purity median from the sequenced samples obtained by PureCN analyses. **(A)** Tumoral purity reported for chemoresistant (left, orange) and chemosensitive (right, blue) NS-TGCT. Each dot represents the tumoral purity of the sample where >0.75 implies high tumoral purity and <0.25 represents low purity. **(B)** Tumoral ploidy reported for chemoresistant (left, orange) and chemosensitive (right, blue) NS-TGCT. Each dot represents the tumoral ploidy of the sample where >1 implies high tumoral ploidy and <1 represents low ploidy.

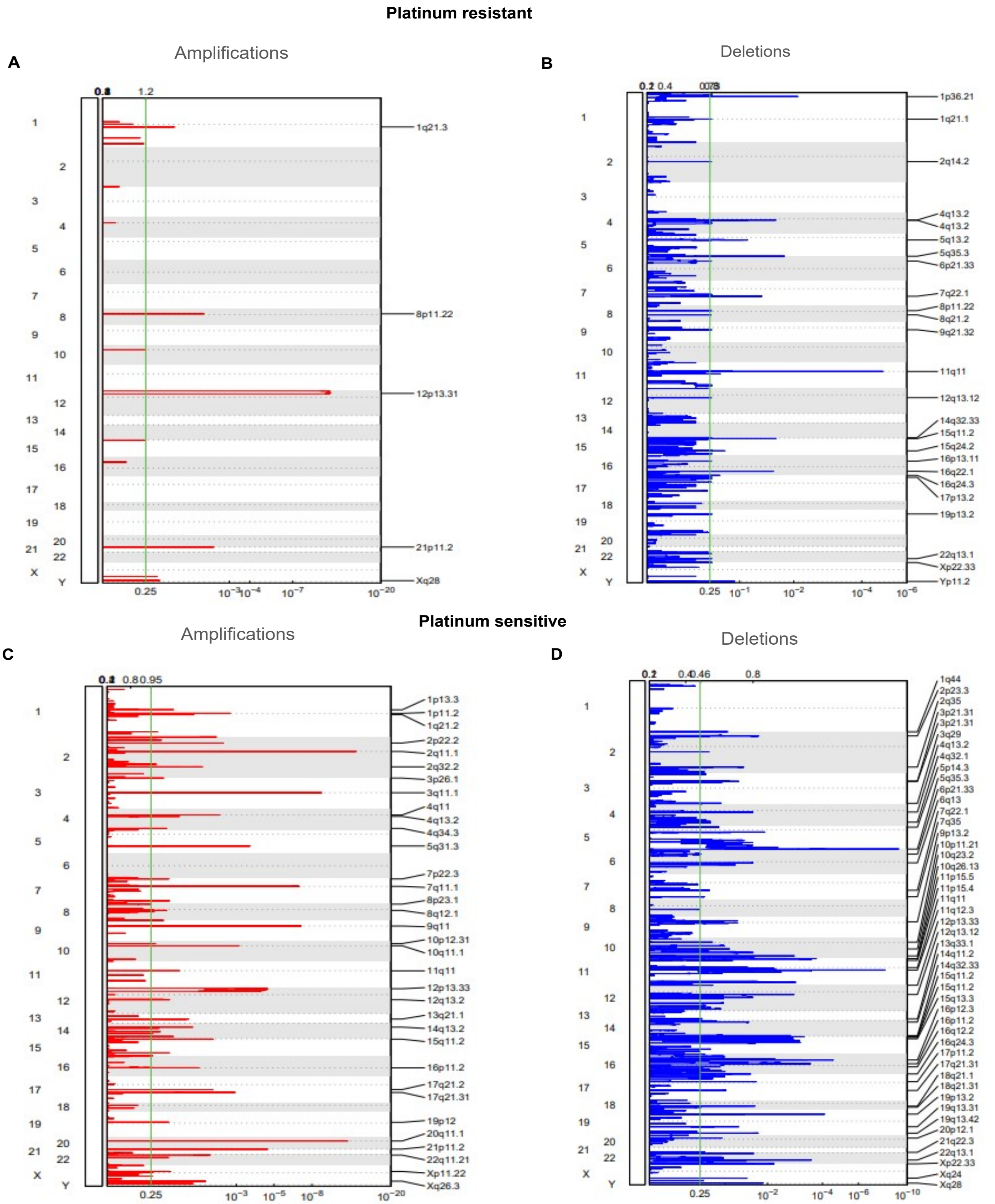

Figure S3. Comparison of amp/del per segment over resistant (up) and sensitive (down) from GISTIC analysis. **(A) & (B)** Amp/Del events (red/blue, respectively) in resistant samples. Left bar represents a total chromosome array, each line indicated are labeled genomic segments and its longitude represents the frequency of each event. G-Score corresponds to the peaks with higher incidences, estimated for gain or loss in those genomic segments. Scheme is the same for **(C) & (D).** Broadly, the GISTIC plot reveals that platinum-sensitive samples have more gain and loss sites than resistant, and copies are lost in different segments more frequently than are lossed.

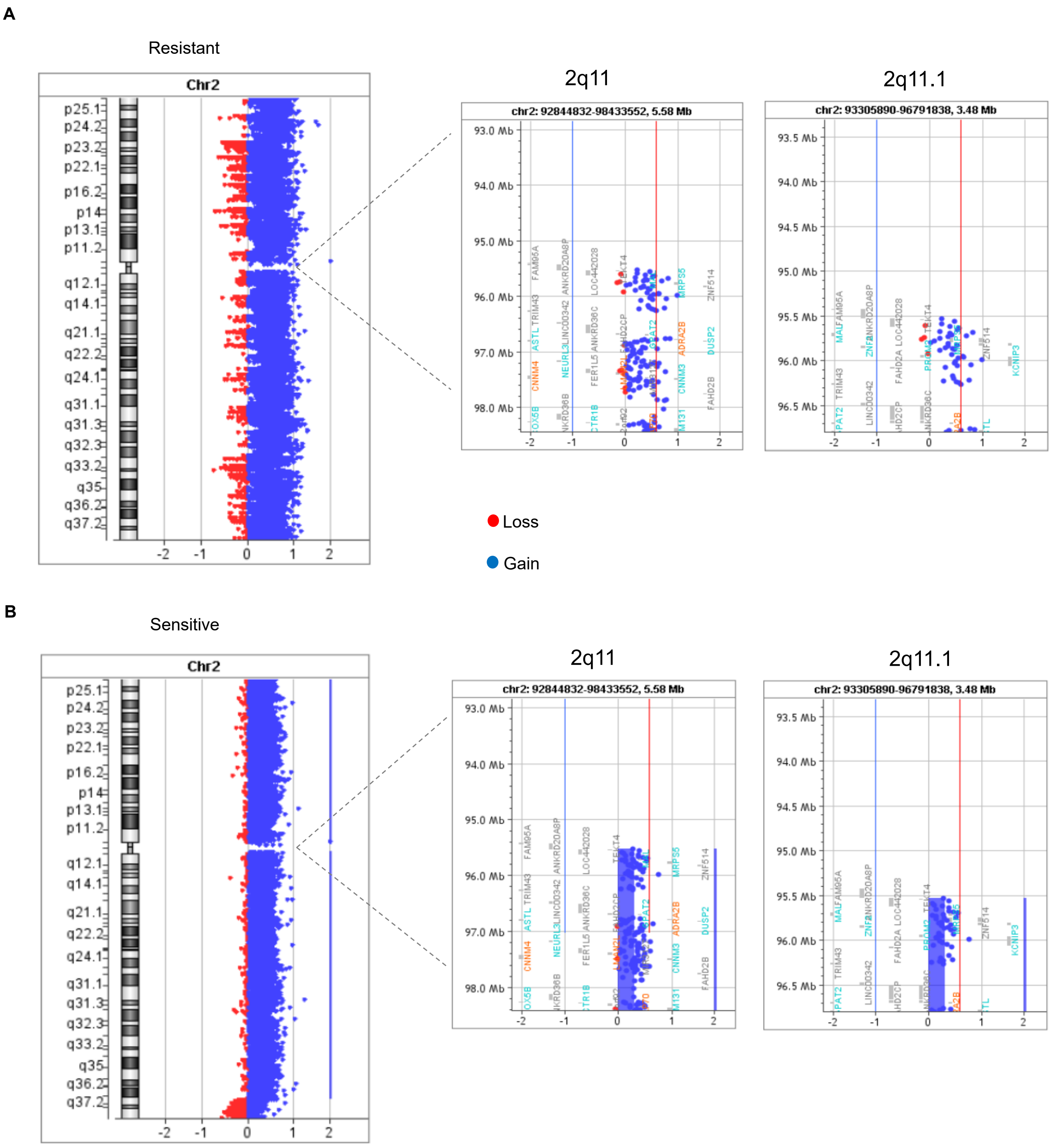

Figure S4. Visualization of aCGH in Chr2 region in two samples to compare focal-CNVs inl 2q11.1 segment. **(A)** A resistant sample (up) included in aCGH, shows the 2q11.1 segment without gains or losses (blue and red, respectively), **(B)** A sensitive sample (down) included in aCGH shows the constant gains in the 2q11.1 segment in sensitive samples. Both **(A)** and **(B)** shows in right a zoom with the genes contained in the 2q11.1 region.
